# Diagnostics for Yaws Eradication: Insights From Direct Next-Generation Sequencing of Cutaneous Strains of *Treponema pallidum*

**DOI:** 10.1093/cid/cix892

**Published:** 2017-10-16

**Authors:** Michael Marks, Maria Fookes, Josef Wagner, Robert Butcher, Rosanna Ghinai, Oliver Sokana, Yaw-Adu Sarkodie, Sheila A Lukehart, Anthony W Solomon, David C W Mabey, Nicholas Thomson

**Affiliations:** 1 Clinical Research Department, Faculty of Infectious and Tropical Diseases, London School of Hygiene and Tropical Medicine, London; 2 Hospital for Tropical Diseases, London; 3 Wellcome Trust Sanger Centre, Cambridge, United Kingdom; 4 Solomon Islands Ministry of Health and Medical Services, Honiara; 5 Kwame Nkrumah University of Science and Technology, Kumasi, Ghana; 6 Departments of Medicine and Global Health, University of Washington, Seattle; 7 Department of Pathogen Molecular Biology, Faculty of Infectious and Tropical Diseases, London School of Hygiene and Tropical Medicine, United Kingdom

**Keywords:** whole-genome sequencing, next-generation sequencing, yaws, *Treponema pallidum*

## Abstract

**Background:**

Yaws-like chronic ulcers can be caused by *Treponema pallidum* subspecies *pertenue*, *Haemophilus ducreyi*, or other, still-undefined bacteria. To permit accurate evaluation of yaws elimination efforts, programmatic use of molecular diagnostics is required. The accuracy and sensitivity of current tools remain unclear because our understanding of *T. pallidum* diversity is limited by the low number of sequenced genomes.

**Methods:**

We tested samples from patients with suspected yaws collected in the Solomon Islands and Ghana. All samples were from patients whose lesions had previously tested negative using the Centers for Disease Control and Prevention (CDC) diagnostic assay in widespread use. However, some of these patients had positive serological assays for yaws on blood. We used direct whole-genome sequencing to identify *T. pallidum* subsp *pertenue* strains missed by the current assay.

**Results:**

From 45 Solomon Islands and 27 Ghanaian samples, 11 were positive for *T. pallidum* DNA using the species-wide quantitative polymerase chain reaction (PCR) assay, from which we obtained 6 previously undetected *T. pallidum* subsp *pertenue* whole-genome sequences. These show that Solomon Islands sequences represent distinct *T. pallidum* subsp *pertenue* clades. These isolates were invisible to the CDC diagnostic PCR assay, due to sequence variation in the primer binding site.

**Conclusions:**

Our data double the number of published *T. pallidum* subsp *pertenue* genomes. We show that Solomon Islands strains are undetectable by the PCR used in many studies and by health ministries. This assay is therefore not adequate for the eradication program. Next-generation genome sequence data are essential for these efforts.

## Teaser

Yaws is an important cause of skin disease in tropical countries. Using next-generation sequencing, we tested yaws-like ulcers, which were negative using standard molecular diagnostics. We found primer binding site mutations explaining the negative results of the standard assay.

Yaws, caused by *Treponema pallidum* subspecies *pertenue*, is a major public health problem in many tropical countries [1]. The disease is most common in West Africa and the Pacific [2] and predominantly affects children living in remote communities. Primary yaws manifests as chronic painless cutaneous ulcers and papillomatous lesions that, if untreated, can progress to cause destructive lesions of bone and soft tissues. In 2012, azithromycin was shown to be a highly effective treatment for yaws [3], prompting a new World Health Organization (WHO) eradication strategy based on community mass treatment with azithromycin [4]. Initial pilot data suggest this is a powerful intervention for reducing transmission within communities [5].

Clinical diagnosis of yaws is unreliable and serological testing is needed to confirm the diagnosis [1, 6, 7]. Serological tests cannot distinguish yaws from syphilis; this distinction relies on consideration of the clinical syndrome, epidemiology, or molecular tools. In many settings, up to 50% of possible yaws lesions are not confirmed serologically [6, 8], reflecting the broad range of causes of skin ulcers in the tropics, with bacterial, fungal, and parasitic infections all being common differential diagnoses. For example, *Haemophilus ducreyi* has been found to be a common cause of chronic ulcerative skin lesions in yaws-endemic areas, and lesions caused by *H. ducreyi* are clinically indistinguishable from yaws [7, 9, 10]. Consequently, WHO recommends molecular diagnostic tests as part of the case definition in yaws eradication efforts [11]. Molecular diagnostics are also used to detect mutations conferring azithromycin resistance [12] and are therefore a central component of the eradication strategy [11].

The genome sequences of *T. pallidum* subspecies are highly conserved, with <0.2% of sequence variation between subspecies [13]. However, there are few complete sequences available to inform selection of molecular targets for polymerase chain reaction (PCR), in part due to our inability to culture *T. pallidum* in vitro. Currently no multiplex assay exists for diagnosis of ulcerative skin lesions in the tropics, but a real-time PCR assay, targeting the *tp0858* gene (a predicted outer membrane protein), has been developed to detect *T. pallidum* subsp *pertenue* and differentiate it from other *T. pallidum* subspecies [8]; this PCR is hereinafter referred to as the 2015 Centers for Disease Control and Prevention (CDC) real-time PCR assay. The assay is believed to have an analytical sensitivity of 10–100 copies per reaction [8]. An alternative approach is to combine a PCR assay targeting a pan-species *T. pallidum* target with secondary PCR for subspecies confirmation [10], but currently the 2015 CDC real-time PCR assay is being used by health ministries to support yaws eradication efforts worldwide. When either of these assays is used, a minority of samples from yaws-like lesions is found to contain *T. pallidum* subsp *pertenue* DNA [7–10].

In a recent study in the Solomon Islands, for example, the seroprevalence of treponemal antibodies was >30% among children aged 5–14 years, and 2% of children in that age group had a skin lesion consistent with active yaws and reactive serological tests, yet no *T. pallidum* subsp *pertenue* DNA was detected using the 2015 CDC real-time PCR assay [9]. Given the strong epidemiological, clinical, and serological evidence of yaws transmission in the Solomon Islands, we hypothesized that this molecular test failed to detect local *T. pallidum* subsp *pertenue* strains. The aim of this study was to use next-generation sequencing to explore the reasons why *T. pallidum* subsp *pertenue* was not detected using the 2015 CDC real-time PCR assay in samples from patients showing clinical and serological evidence of yaws, and to develop a modified assay capable of detecting these missed samples.

## METHODS

### Sample Collection

Samples were collected during surveys conducted in the Solomon Islands in 2013 and Ghana in 2014 [6, 7]. In brief, the survey in the Solomon Islands was a community-based cluster randomized prevalence survey, whereas the survey in Ghana used school-based recruitment. In both surveys, lesion swabs or lesion crusts were collected from individuals with chronic ulcerative lesions clinically consistent with yaws. DNA was prepared from residual sample material from these 2 original surveys.

### Data Collection

Clinical and demographic data for this study were limited to data collected in the original surveys [6, 7, 9], which included age, gender, lesion location, and serological results. In Ghana, ulcer samples had been collected directly onto dry Dacron swabs. In the Solomon Islands, swab exudate was placed either into transport medium (AssayAssure, Sierra Molecular) or onto an FTA Elute Card (Whatman). In both studies, where lesion crusts were present, these were removed and placed in transport medium (AssayAssure). As part of the original surveys, all individuals had a serum sample tested using a *T. pallidum* particle agglutination assay and, when this was positive, a quantitative rapid plasma reagin assay [7, 9]. Lesion samples had been assayed with the 2015 CDC real-time PCR assay for *T. pallidum* subsp *pertenue* [7] as part of the earlier studies, and the results were available for this study.

### Sample Preparation

Following collection, samples were stored at –20**°**C in Ghana and the Solomon Islands, before being transported to the CDC on dry ice. Following this, samples were stored again at –20**°**C before being transported back to the London School of Hygiene and Tropical Medicine on dry ice for the current study. DNA was extracted from the residual sample material using the QIAamp Mini kit from Qiagen with variations (Supplementary Appendix 1). DNA preparations were screened for the presence of *T. pallidum* using a quantitative PCR (qPCR) assay targeting the *tp47* (*tp0574*) gene (hereafter “*tp47*-PCR”) that is conserved in all known members of the *T. pallidum* species, but which does not distinguish between *T. pallidum* subspecies [14, 15]. For *T. pallidum*–positive samples, we used a DNA concentration cutoff of 10 copies/μL to select samples likely to yield accurate whole genomes. Samples above this cutoff were sent for direct (non-culture-based) sequencing.

### Whole-Genome Sequencing and Analysis

*Treponema pallidum* genomic DNA (gDNA) was quantified, per sample, and supplemented with human gDNA (Invitrogen) to make a final total DNA concentration of 500 ng. DNA samples were fragmented to an average size of 150 bp and subjected to DNA library creation using established Illumina paired-end protocols [16]. Adapter-ligated libraries were amplified and indexed via PCR. A portion of each library was used to create an equimolar DNA pool. Each pool was hybridized to custom-made SureSelect RNA baits (Agilent Technologies; baits based on all published sequences of *T. pallidum* and *Treponema paraluiscuniculi* genomes [17–19]) (Supplementary Appendix 1). Targets were captured and amplified in accordance with manufacturer recommendations. Enriched libraries were subjected to standard 75-bp paired-end sequencing (HiSeq 2000; Illumina) following manufacturer instructions. Samples’ public accession numbers are listed in Supplementary Table 1.

**Figure 1.**
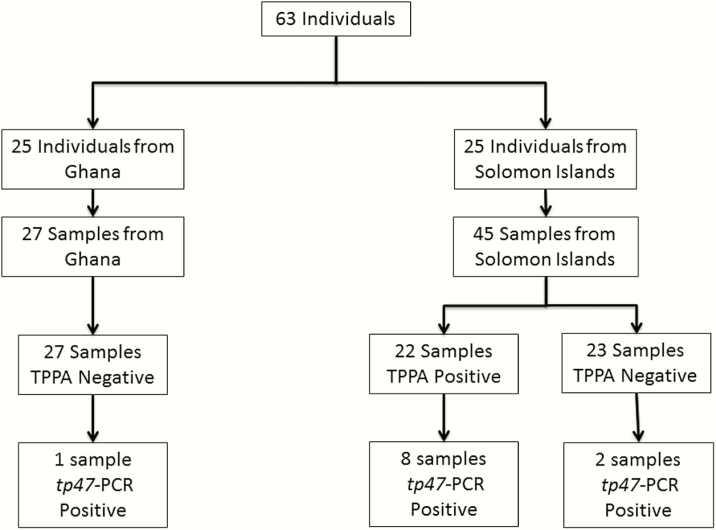
Study flowchart. Samples were originally collected in 2 studies conducted in Ghana and the Solomon Islands. The results of *Treponema pallidum* particle agglutination assays conducted in the original studies and of the *tp47* polymerase chain reaction assay performed in this study are shown. Abbreviations: PCR, polymerase chain reaction; TPPA, *Treponema pallidum* particle agglutination.

We used whole-genome sequencing (WGS) read data to estimate phylogenies for *T. pallidum* subsp *pertenue*: reads were mapped using SMALT [20] to the reference genome *T. pallidum* subsp *pertenue* strain Samoa D (EMBL CP002374 [21]). Single-nucleotide polymorphisms (SNPs) were identified for each mapped sample using SamTools [22] with previously described settings [23]. Gubbins was used (with default settings; see Supplementary Appendix 1 for more details) to identify recombination blocks (defined by high SNP density) using the whole-genome SNP data of each sample as previously described [24]. SNPs in recombination blocks were excluded from phylogenetic analysis (all recombination blocks are listed in Supplementary Table 2) as they do not represent the underlying phylogeny of the host (Figure 1). The remaining chromosomal SNPs from each isolate were used to generate a multiple alignment of concatenated SNPs for all isolates. Maximum likelihood phylogenetic trees were estimated using RAxML with a general time reversible site model, using gamma correction for among-site variation and 100 bootstrap replicates [25]. De novo genome assemblies were performed as previously described [26] or using SPAdes [27]. Contigs were automatically annotated using in-house pipelines as previously described [28]. Default parameters for software packages are described in Supplementary Appendix 1. Genes of interest were identified and curated by hand in pairwise comparisons with the reference genome using ACT or Artemis [29]. Mapping and assembly statistics are listed in Supplementary Table 1. Supplementary Table 1 also includes data for simulated reassemblies of the 2 reference sequences to determine the achievable reassembly size from Illumina 75-bp paired-end reads and the standard assembly protocols used in this study. The 75-bp paired-end reads were generated in silico from the reference genome using in-house scripts to simulate raw Illumina WGS data.

### Development of a Modified PCR Assay

**Figure 2.**
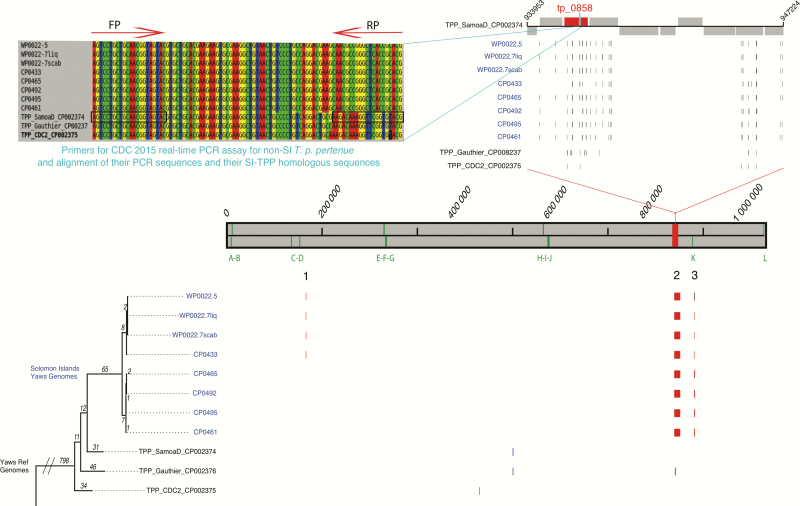
Phylogenetic tree of *Treponema pallidum* subspecies *pertenue* genomes. Phylogenetic tree of *T. pallidum* subsp *pertenue* sequences estimated by mapping to the *T. pallidum* subsp*. pertenue* SamoaD reference genome. Recombination blocks shared by 1 or more isolates are numbered and those unique to a single isolate are unnumbered. Vertical bars over the genome length (gray block) mark the location of the *tpr* genes. Coordinates are in base pairs with respect to the reference gnome. Recombination blocks present in the Solomon Islands *T. pallidum* subsp *pertenue* isolates are numbered (1–3; main frame). Recombination block 2 spans several genes (top right), including *Tp_0858*, which contains the primer-binding sites used in the 2015 Centers for Disease Control and Prevention (CDC) real-time polymerase chain reaction (PCR) assay. The positions of single-nucleotide polymorphisms identified in region 2 are shown for each isolate (top right) compared to the reference genome. Top left shows an alignment of the region of the reference genomes amplified by the 2015 CDC real-time PCR assay for *T. pallidum* subsp *pertenue* isolates compared to the same region in samples from the Solomon Islands. Primer binding sites are indicated by arrows. Several mutations within these sites are found in Solomon Islands samples. Abbreviations: CDC, Centers for Disease Control and Prevention; FP, forward primer; PCR, polymerase chain reaction; RP, reverse primer; SI, Solomon Islands; TPP, *Treponema pallidum* subsp *pertenue*.

**Figure 3.**
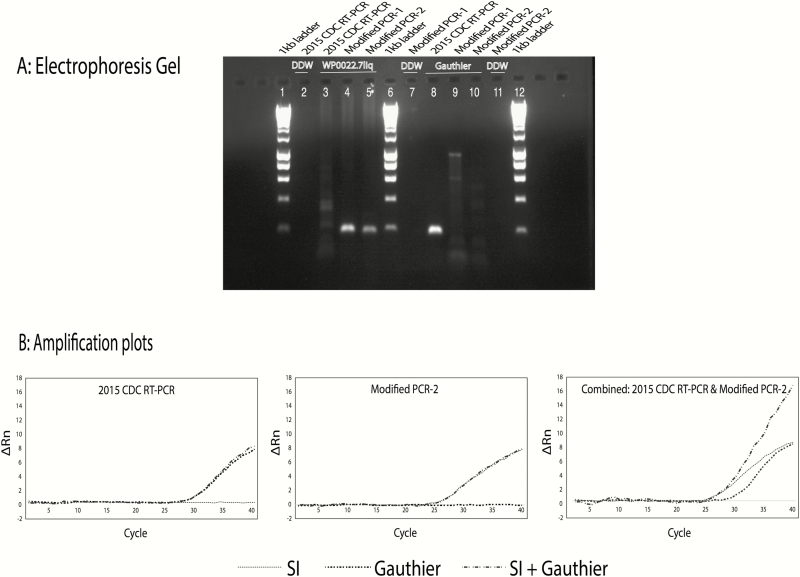
Gel and quantitative polymerase chain reaction (PCR) assays utilizing both existing and new PCR primers. *A*, Gel picture showing PCR products for the genomic DNA (gDNA) of a Solomon Islands (SI) sample WP0022.7liq, and a non-SI sample, *Treponema pallidum* subsp *pertenue* Gauthier, with the use of the generic forward and reverse primers as indicated. The reverse primer of the Centers for Disease Control and Prevention (CDC) 2015 real-time (RT) PCR assay is labeled reverse primer in Figure 2 . Forward primer (2015 CDC RT-PCR): 5ʹ-CGGCCACCAACTTGGGATTGAC-3ʹ. Reverse primer (2015 CDC RT-PCR): 5ʹ-CGTACACCGAACCTTTGTCTT-3ʹ. Reverse primer (modified PCR-1): 5ʹ- GTGCGGTGAGCCCGGCGTT-3ʹ. Reverse primer (modified PCR-2): 5ʹ-GTGAGCCCGGCGTT-3ʹ. *B*, Quantitative PCR amplification curves of new PCR products obtained for the gDNA of the non-SI sample, an SI sample, and both samples (all as in *A*) combined by using specific reverse primers. Forward primer (2015 CDC RT-PCR): 5ʹ-CGGCCACCAACTTGGGATTGAC-3ʹ. Probe: 5ʹ-FAM-GCTGCAAGGAGAAGTCCTGCTGC-TAMRA-3ʹ. Reverse primer (2015 CDC real-time PCR): 5ʹ-CGTACACCGAACCTTTGTCTT-3ʹ. Reverse primer (modified PCR-2): 5ʹ-GTGAGCCCGGCGTT-3ʹ. Abbreviations: ∆Rn, normalized reporter signal; CDC, Centers for Disease Control and Prevention; DDW, double distilled water; RT-PCR, real-time polymerase chain reaction; SI, Solomon Islands.

We used results of WGS to design a modified PCR assay targeting the *tp0858* gene. We designed 2 new reverse PCR primers with different lengths and annealing temperatures, denoted “modified PCR-1” (5ʹ-GTGCGGTGAGCCCGGCGTT-3ʹ) and “modified PCR-2” (5ʹ-GTGAGCCCGGCGTT-3ʹ). We tested the assay using both gDNA from a Solomon Islands sample (WP0022.7liq) and from a non–Solomon Islands sample (non-SI, strain Gauthier). Double-distilled water was used as a control. We selected the shorter modified PCR-2 reverse primer for a modified qPCR assay. We performed qPCR using the original forward and reverse (denoted “2015 CDC RT-PCR” in Figure 2) 2015 CDC real-time PCR primers [8], as well as the original forward primer plus the modified PCR-2 reverse primer (denoted “modified PCR-2” in Figure 2). We also used the forward primer plus both reverse primer variants together (Figure 3; for conditions, see Supplementary Appendix). The PCR was run on an Applied Biosystems StepOne Plus qPCR machine using the TaqMan Fast Advanced Master Mix. We used a probe that detects both Solomon Islands and non–Solomon Islands strains (5ʹ-FAM-GCTGCAAGGAGAAGTCCTGCTGC-TAMRA-3ʹ).

### Ethics Approval

The studies were approved by the London School of Hygiene and Tropical Medicine, Solomon Islands National Health Research, and Kwame Nkrumah University of Science and Technology ethics committees.

## RESULTS

### Participants

Seventy-two residual samples taken from 63 people were included in this study [6, 7, 9] (Figure 1). The median age of participants in the original studies was 9 years (interquartile range, 7–11 years) and 36 contributing participants (57.1%) were male. Of samples recovered and tested here, 69 were collected from lesions located on the leg; lesion location was not recorded for 3 samples [6, 7, 9]. In the original studies [7, 9], none of the samples from either site had tested positive for *T. pallidum* subsp *pertenue* using the 2015 CDC real-time PCR assay, but 24 samples had tested positive for *H. ducreyi* using a 16S ribosomal RNA targeted PCR assay (15 from the Solomon Islands and 9 from Ghana).

Using the screening *tp47*-PCR assay, 11 samples were shown to be positive for *T. pallidum* (Figure 1). In 10 *tp47*-PCR–positive samples from the Solomon Islands, the median *T. pallidum* chromosomal copy number was 1084/μL. The single Ghanaian sample that was *T. pallidum* positive using the *tp47*-PCR assay had the lowest *T. pallidum* chromosomal copy number (~38/μL) of any positive sample included in this study.

### Direct WGS of *T. pallidum* subsp *pertenue*

From 11 *T. pallidum–*positive samples, 8 (72.7%) complete genomes of *T. pallidum* subsp *pertenue* were obtained. The 8 genomes were generated from samples taken from 6 individuals in the Solomon Islands, including 3 samples collected from the same individual (Supplementary Table 1). There was no evidence of sequence heterozygosity in any of the Solomon Islands *T. pallidum* subsp *pertenue* genomes to indicate that any individual subject was simultaneously infected with multiple strains of *T. pallidum* subsp *pertenue.*

### *T. pallidum* subsp *pertenue* Genome Analysis

Phylogenetic analysis demonstrated that *T. pallidum* subsp *pertenue* strains from the Solomon Islands form a discrete lineage that can be further subdivided into 2 distinct clades, both of which are distinct from all previously sequenced *T. pallidum* subsp *pertenue* samples. Solomon Islands *T. pallidum* subsp *pertenue* genomes were highly conserved, separated by a maximum pairwise distance of <20 SNPs (Figure 2). There were no whole gene differences among the manually curated Solomon Islands genomes or when comparing the Solomon Islands genomes to the reference sequence (SamoaD strain) or to previously published sequences. Three genomic regions were identified as recombinant in all, or a subset, of the Solomon Islands *T. pallidum* subsp *pertenue* genomes (Figure 2). Thirteen recombinant regions were defined, across all subspecies included, due to their high SNP density (Figure 2 and Supplementary Table 2). Recombinant region 1 included the *TPESAMD_0134* gene predicted to encode a putative outer member protein [21] (sequence accession number CP002374). Region 2 was predicted to encode 10 genes and 2 gene remnants, including *tp0858*. Region 3 encompassed the *tprK* gene, which is known in *T. pallidum* subsp *pallidum* to undergo antigenic variation via segmental gene conversion. No 23S rDNA mutations known to confer resistance to azithromycin were found.

The increased SNP density found in region 2 included several variations within the 2015 CDC real-time PCR assay reverse primer-binding site (within *tp0858*) for (Figure 2). The forward 2015 CDC real-time PCR primer-binding site is conserved in all *T. pallidum* subsp *pertenue* isolates (Figure 2). Although variation within *tp0858* is seen when comparing the Solomon Islands *T. pallidum* subsp *pertenue* isolates’ sequences to the reference (Figure 2), this likely recombination block sequence is fixed and conserved in all of the Solomon Islands isolates we sequenced. This suggests that this sequence is representative of isolates circulating in the South Pacific. These data also provide an explanation as to why the 2015 CDC real-time PCR assay had failed in this setting (Figure 3).

### Modified PCR Assay

Using our modified PCR assay, we were able to amplify DNA from Solomon Islands samples (Figure 3A) and demonstrate that the Solomon Islands new reverse primer (but not the existing CDC real-time PCR reverse primers) detect *T. pallidum* subsp *pertenue* samples from the Solomon Islands (Figure 3B). These data also show that these primers can be used successfully in combination (Figure 3).

## DISCUSSION

In this study we aimed to explain why, in a country endemic for yaws, all previously tested samples had tested negative for *T. pallidum* subsp *pertenue* DNA using the standard 2015 CDC real-time PCR assay [9]. We identified a large number of *T. pallidum* subsp *pertenue* positive samples that were missed using the 2015 CDC real-time PCR assay. These all demonstrated mutations in the PCR primer-binding sites targeted by that assay. PCR of these samples was negative when the 2015 CDC real-time PCR primers were used but positive for *T. pallidum* subsp *pertenue* using a modified assay based on the sequence data generated in this study (Figure 3). Overall, 22% of samples from the Solomon Islands were positive using our approach, a proportion comparable to that seen in other studies in Papua New Guinea and Vanuatu [8, 10]. The current data call in to question the validity of the finding that many yaws-like lesions are negative on molecular testing using the 2015 CDC real-time PCR assay [7–10]. It is plausible that next-generation sequencing might identify further *T. pallidum* subsp *pertenue* strains from other contexts that test negative by the 2015 CDC real-time PCR assay.

Because lesions caused by a variety of other bacteria appear clinically similar to yaws, molecular diagnostics have become key to the case definition in yaws eradication programs [11]. The findings presented here demonstrate that the 2015 CDC real-time PCR assay does not detect all *T. pallidum* subsp *pertenue* isolates. Given our findings, it may be technically challenging to design a single-target *T. pallidum* subsp *pertenue–*specific PCR. We believe a strategy using a PCR that targets conserved regions of *T. pallidum* would be more appropriate and less prone to false-negative results. If required, additional testing using a panel of PCR assays for subspecies-level identification could be performed (eg, in cases of genital lesions or older individuals where syphilis is a differential diagnosis). Assays suitable for this approach exist [10], although it is not certain that they will not also miss as-yet-unrecognized *T. pallidum* subsp *pertenue* strains were variation to exist in the *tp47* gene targeted by the screening PCR. Sequencing of a larger number of samples would help confirm that the *tp47* gene is highly conserved and therefore a suitable target for screening PCR.

Understanding of treponemal diversity has been severely hampered by our inability to culture *T. pallidum* in vitro. The direct sequencing techniques used in this study may provide insights into unanswered questions about yaws. The yaws clinical phenotype varies between West Africa, where papillomatous lesions predominate, and the Pacific, where ulcerative lesions are more common. Next-generation sequencing may help to reveal contributing causes. Even in the absence of known mutations associated with azithromycin resistance, some patients do not experience cure following treatment. The techniques used in this study may allow characterization of pathogen factors associated with outcome. Multicountry studies to explore these questions are warranted.

Although the *T. pallidum* subspecies that cause syphilis and yaws are extremely closely related [13], the phenotypes of the diseases differ. There is limited understanding of the factors driving observed differences in tropism and virulence between subspecies. Neurological and cardiovascular manifestations are classical features of tertiary syphilis, yet are seen in a minority of untreated patients. These severe clinical manifestations are generally believed to be absent in yaws, although older studies in Ghana noted that cardiovascular manifestations might occasionally present [30], and some studies have suggested that neurological manifestations and congenital transmission may occur in yaws [31]. Examination of larger numbers of treponemal genomes by direct sequencing might help us understand the difference in outcomes of these infections.

A limitation of our study is the relatively small number of samples sequenced. However, the *T. pallidum* subsp *pertenue* genome sequences obtained in this study double the total number of described sequences available, including data from all previous studies and have provided new insights into *T. pallidum* subsp *pertenue* diversity and the design of molecular diagnostics. It is possible that repeat freeze-thawing and use of residual sample may have limited the amount of genomic material available for sequencing in the current study. One sample from Ghana had a positive *tp47*-PCR assay despite negative serology. Although the overall chromosomal copy number was low, we believe this is consistent with early active yaws before a serological response has occurred (analogous to early syphilis).

In this study, we demonstrate the clinical and public health value of next-generation sequencing techniques in yaws. These techniques could provide valuable data on the etiology of yaws and yaws-like lesions. We identified a high proportion of lesions containing a new strain of *T. pallidum* subsp *pertenue* undetectable by the 2015 CDC real-time PCR assay. Importantly, our findings demonstrate that this assay will not be adequate for use in yaws eradication efforts. Urgent steps are needed to improve our understanding of *T. pallidum* subsp *pertenue* diversity worldwide, to ensure molecular diagnostics are program ready.

## Supplementary Data

Supplementary materials are available at *Clinical Infectious Diseases* online. Consisting of data provided by the authors to benefit the reader, the posted materials are not copyedited and are the sole responsibility of the authors, so questions or comments should be addressed to the corresponding author.

## Supplementary Material

Supplementary Figure S1

Supplementary Table S1

Supplementary Table S2

Supplementary Documents

Supplementary Appendix

## Notes

***Author contributions.*** M. M., M. F., and N. T. wrote the first draft of the manuscript. M. F., J. W., and R. B. conducted laboratory work and analyzed the data. M. M., R. G., O. S., and Y. A. S. conducted fieldwork. M. M., S. A. L., A. W. S., D. C. W. M., and N. T. designed the study and analyzed the data. All authors revised the manuscript.

***Financial support.*** M. M. is supported by the Wellcome Trust (grant number 102807). N. R. T. and M. C. F. were supported by the Wellcome Trust (grant number 098051). Funding for the fieldwork in Ghana was additionally supported by a grant from the Royal Society of Tropical Medicine and Hygiene (to M. M.).

***Potential conflicts of interest.*** All authors: No reported conflicts of interest. All authors have submitted the ICMJE Form for Disclosure of Potential Conflicts of Interest. Conflicts that the editors consider relevant to the content of the manuscript have been disclosed.
