# Supplementary figures and images for: Diagnostics for Yaws Eradication: Insights From Direct Next-Generation Sequencing of Cutaneous Strains of Treponema pallidum
Source: Clin Infect Dis. 2017 Oct 16;66(6):818–24. doi: 10.1093/cid/cix892 (PMC5848336; doi:10.1093/cid/cix892)

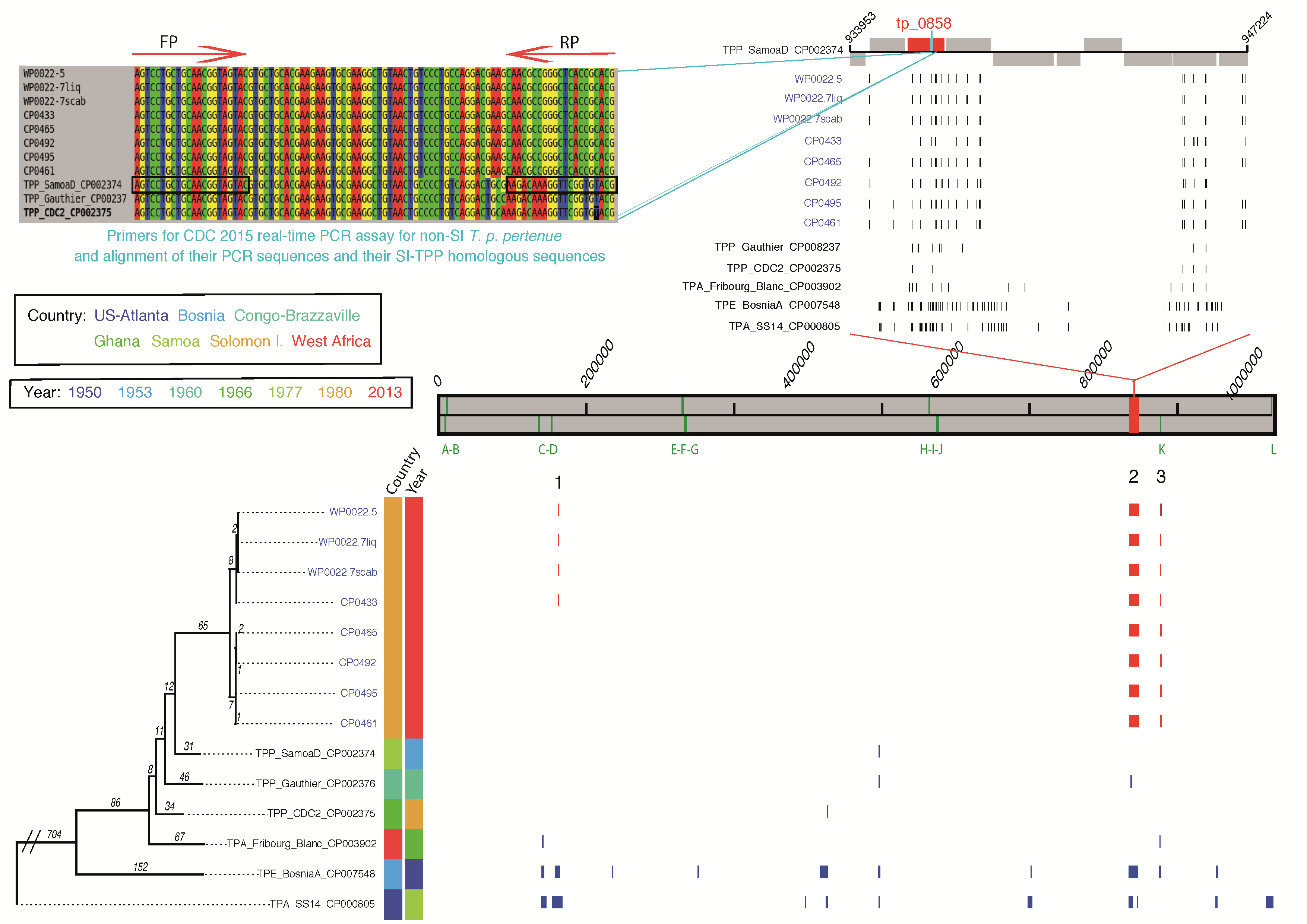

Supplement: Supplementary Figure S1 [file cix892_suppl_supplementary_figure_s1.png]
