# Supplementary material for: Diagnostics for Yaws Eradication: Insights From Direct Next-Generation Sequencing of Cutaneous Strains of Treponema pallidum
Source: Clin Infect Dis. 2017 Oct 16;66(6):818–24. doi: 10.1093/cid/cix892 (PMC5848336; doi:10.1093/cid/cix892)
Supplement: Supplementary Documents [file cix892_suppl_supplementary_documents.docx]

**List of Supplementary Documents:**

Supplementary Appendix

Supplementary Appendix 1 – Sample processing and handling methods

Supplementary Tables

Supplementary Table 1 - Accession numbers and coverage of whole genome sequences

Supplementary Table 2 – Regions of high density SNPs, including recombination for *T. p.* subsp. *pertenue* sequences
