# Supplementary material for: Diagnostics for Yaws Eradication: Insights From Direct Next-Generation Sequencing of Cutaneous Strains of Treponema pallidum
Source: Clin Infect Dis. 2017 Oct 16;66(6):818–24. doi: 10.1093/cid/cix892 (PMC5848336; doi:10.1093/cid/cix892)
Supplement: Supplementary Appendix [file cix892_suppl_supplementary_appendix.docx]

**Supplementary Appendix 1**

**DNA Extraction Methods Used**

Dry Dacron swab tips from Ghana were suspended in 1 ml of lysis buffer (10 mM Tris, pH=8.0; 0.1M EDTA, pH=8.0; 0.5% SDS), vortexed vigorously for 5 min and the supernatants divided into three tubes. The Qiagen buccal swab spin protocol (which includes a Proteinase K reaction step) was then followed. The same procedure was applied to Solomon Islands samples (transported in AssayAssure transport buffer, Sierra Molecular); lesion crusts were suspended in the liquid buffer, were pelleted and DNA extractions were performed on both the liquid phase (as above) and pellet. DNA extraction of the lesion pellet was performed as follows: pellets were re-suspended in 500 ml of lysis buffer, treated with proteinase K and transferred to a Lysing Matrix E tube (MP Biomedicals) prefilled with various sized glass beads. 200 ml of AL buffer was added to this. Filled tubes were homogenized for 40secs at speed 6000 (FastPrep Homogenizer, MP Biomedicals). The tubes were then centrifuged for 10min at 13,200rpm and the Qiagen DNA extraction protocol followed as above. The DNA was then extracted according to manufacturers instructions. Each DNA specimen was eluted in 150μl AE buffer.

**SureSelect Probe Design**

*Treponema* *pallidum* species wide array was constructed to be both specific and pull-down DNA from a range or relevant *Treponema* genomes. This array was constructed iteratively using *Treponema* *pallidum* subsp. *pallidum* str. Nichols (EMBL: AE000520) as a base sequence, to which additional variant (see below) or novel sequences, from available published genomes (EMBL accession numbers CP000805, *Treponema* *pallidum* subsp. *pallidum* str SS14; CP003115, *Treponema* *pallidum* subsp. *pallidum* str DAL-1; CP002376, *Treponema* *pallidum* subsp. *pertenue* str. Gauthier; and CP002103, *Treponema* *paraluiscuniculi* str Cuniculi A), were added iteratively to build a non-redundant *Treponema pallidum* and *Treponema paraluiscuniculi* pan genome pull-down array. Sequences were only added to the base pan genome sequence if they were novel or variant: Novel sequences were defined as regions that were absent from the growing pan genome consensus sequence. Variant sequences showed less than 75% sequence identity to sequences already in the consensus sequence. Agilent 120 bp RNA baits were designed to cover the pan-genome consensus sequence to a depth of 3X. RNA baits were screened against the pan-genome consensus sequence for redundancy (see http://earray.chem.agilent.com/suredesign). Adding additional non-*Treponema pallidum* subsp. *pertenue* sequences maximises the utility of the array without compromising its specificity. All captured DNA was validated by sequencing and mapping to an appropriate reference genome.

**Bioinformatic Settings**

Base-calling required agreement of 75% of reads (minimum read depth >30). FASTQ files used Illumina 1.8 +33 quality encoding. We used standard settings for each software package used to perform our bio-informatic analysis. The identification of recombination blocks, using Gubbins ^1^, required a minimum of 3 base-substitutions to define a possible recombination block. For the generation of Maximum likelihood phylogenetic trees withing Gubbins, RAxML was used^2^; we performed a minimum of five iterations and ceased performing further iterations based on weighted Robinson-Foulds criteria^3^. *De novo* genome assemblies were performed as previously described^4^ or using SPAdes^5^. For the latter, the single cell mode and minimize options were selected, which minimizes the number of mismatches and short INDELS when generating alignments. For the assemblies KMER sizes of 21, 33 and 55 were tested.

**qPCR Conditions**

All qPCR experiments were run with conditions: 50℃ for 2 min then 95℃ for 20 sec followed by 40 cycles of 95℃ for 1 sec and 60℃ for 20 sec. Results are presented in **Figure 2,** Panel B).

**Supplementary References**

1 Croucher NJ, Page AJ, Connor TR, *et al.* Rapid phylogenetic analysis of large samples of recombinant bacterial whole genome sequences using Gubbins. *Nucleic Acids Res* 2015; **43**: e15.

2 Stamatakis A. RAxML-VI-HPC: maximum likelihood-based phylogenetic analyses with thousands of taxa and mixed models. *Bioinformatics* 2006; **22**: 2688–90.

3 Robinson DF, Foulds LR. Comparison of phylogenetic trees. *Math Biosci* 1981; **53**: 131–47.

4 Bronowski C, Fookes MC, Gilderthorp R, *et al.* Genomic characterisation of invasive non-typhoidal Salmonella enterica Subspecies enterica Serovar Bovismorbificans isolates from Malawi. *PLoS Negl Trop Dis* 2013; **7**: e2557.

5 Bankevich A, Nurk S, Antipov D, *et al.* SPAdes: a new genome assembly algorithm and its applications to single-cell sequencing. *J Comput Biol J Comput Mol Cell Biol* 2012; **19**: 455–77.
